# Molecular and Clinical Characterization of Crimean–Congo Hemorrhagic Fever in Bulgaria, 2015–2024

**DOI:** 10.3390/pathogens14080785

**Published:** 2025-08-06

**Authors:** Kim Ngoc, Ivan Stoikov, Ivelina Trifonova, Elitsa Panayotova, Evgenia Taseva, Iva Trifonova, Iva Christova

**Affiliations:** 1 Department of Microbiology, National Center of Infectious and Parasitic Diseases, 1504 Sofia, Bulgaria; kim.ngoc@ncipd.org (K.N.);; 2 Department of Virology, National Center of Infectious and Parasitic Diseases, 1233 Sofia, Bulgaria

**Keywords:** Crimean–Congo hemorrhagic fever, Bulgaria, tick-borne infections

## Abstract

Crimean–Congo hemorrhagic fever (CCHF) is a zoonotic viral disease endemic to parts of Africa, Asia and southeastern Europe. Bulgaria is one of the few European countries with the consistent annual reporting of human CCHF cases. This study provides a descriptive overview of 24 confirmed CCHF cases in Bulgaria between 2015 and 2024. Laboratory confirmation was performed by an enzyme-linked immunosorbent assay (ELISA) and/or real-time reverse transcriptase polymerase chain reaction (RT-qPCR) testing. Common findings included fever, fatigue, gastrointestinal symptoms, thrombocytopenia, leukopenia, liver dysfunction and coagulopathy. Two fatal cases were recorded. Two samples collected in 2016 and 2024 were subjected to whole-genome sequencing. Phylogenetic analysis showed that both strains clustered within the Turkish branch of the Europe 1 genotype and shared high genetic similarity with previous Bulgarian strains, as well as strains from neighboring countries. These findings suggest the long-term persistence of a genetically stable viral lineage in the region. Continuous molecular and clinical surveillance is necessary to monitor the evolution and public health impact of CCHFV in endemic areas.

## 1. Introduction

Crimean–Congo hemorrhagic fever (CCHF) is a zoonotic viral disease caused by the Crimean–Congo hemorrhagic fever virus (CCHFV), a member of the *Orthonairovirus* genus in the family *Nairoviridae* [1,2]. Its main vector and reservoir are *Hyalomma* spp. ticks [3,4], but it has also been detected in ticks belonging to the genera *Dermacentor*, *Rhipicephalus*, *Haemaphysalis*, and *Amblyomma* [5,6]. Transmission to humans occurs mainly through tick bites or contact with the blood or tissues from infected animals [7,8]. Human-to-human transmission has also been reported, particularly in healthcare settings [9]. The risk of infection is highest among individuals with frequent exposure to livestock or ticks, including farmers, butchers, veterinarians, and healthcare workers [10,11]. Clinical disease in humans can range from a mild febrile illness to severe hemorrhagic disease with multiorgan failure and death [2,6,12]. Reported case fatality rates vary in a wide range (from under 5% to over 40%) and are thought to be influenced by a combination of host and viral factors, as well as regional healthcare infrastructure [8,10]. Genotypic variation among circulating strains may contribute to differences in disease severity across endemic regions [11,12].

CCHFV is endemic in regions across Africa, the Middle East, Asia, and southeastern Europe [1,13]. Viral adaptation to local hosts and environmental conditions has led to the emergence of six genetic lineages or clades based on the S segment—three in Africa (Africa 1–3), two in Asia (Asia 1–2), and one in Europe (Europe 1), each generally corresponding to the region where it was first identified [10,12]. A former seventh lineage, Europe 2 (also known as AP92-like), has recently been reclassified as a distinct species, *Orthonairovirus* parahaemorrhagie [14]. Members of the same clade can originate from geographically distant regions, likely due to the long-distance spread of infected ticks by migratory birds or the international shipment of livestock. When introduced into new ecological niches, the virus can become established and maintained in local tick and animal populations [1,15]. 

By some estimations, CCHFV was introduced into Europe a few hundred years ago via the Volga Delta region and from there has spread across the Balkans, where it remains endemic [16]. In the region, Bulgaria has had one of the longest-standing histories with CCHF, with cases being reported regularly over the past 70 years [17,18,19]. Earlier phylogenetic studies have shown that Bulgarian strains predominantly cluster within the Europe 1 lineage. However, sequences corresponding to the Europe 2 lineage, now classified as *O. parahaemorrhagie*, have also been detected in ticks from Bulgaria, particularly in the southeastern districts [17].

In recent years, climate change and global trade have raised concerns about the geographical expansion of *Hyalomma* tick populations—modeling studies have indicated that areas of Europe currently free of CCHF may offer suitable environments for the establishment of *Hyalomma* ticks and subsequent introduction of CCHFV [10,20,21]. Continuous surveillance remains critical for understanding the clinical features of the disease and the molecular evolution of the virus, particularly in endemic regions like Bulgaria, which is one of the few European countries with regular autochthonous cases. The aim of this study is to provide a descriptive overview of laboratory-confirmed CCHF cases in Bulgaria between 2015 and 2024. In addition, we report two previously unpublished genome sequences of CCHFV and explore their relationship with other known strains in the region.

## 2. Materials and Methods

### 2.1. Patient Data and Sample Collection

Between 2015 and 2024, blood samples from patients with suspected Crimean–Congo hemorrhagic fever (CCHF) were submitted to the National Reference Laboratory for Vector-Borne Infections in Sofia, Bulgaria, as part of routine diagnostic investigations. The specimens included either whole blood or serum samples, depending on local hospital procedures. The samples were referred by hospitals across the country based on the clinical and epidemiological suspicion of CCHF. Each sample was accompanied by a brief clinical record including basic epidemiological data, clinical presentation, and available paraclinical test results (e.g., hematological, biochemical, and coagulation parameters). During the study period, approximately 300 samples from suspected CCHF cases were tested, of which 24 were laboratory-confirmed and included in the analysis.

### 2.2. Case Definition

In accordance with Ordinance 21/18.07.2005 on the procedure for the registration, notification, and reporting of communicable diseases in Bulgaria [22], the case definition for CCHF includes clinical, laboratory and epidemiological criteria. The clinical criteria include sudden onset with high fever and chills, myalgia, nausea, anorexia, vomiting, headache, pain in the lower back and hemorrhagic manifestations. The laboratory criteria include virus isolation, the detection of CCHFV RNA, and/or a positive serological test for CCHFV-specific antibodies. The epidemiological criteria require the presence of at least one relevant exposure, such as contact with animals, a history of tick exposure, contact with an infected person or a common source of infection. Based on these criteria, a probable case is any individual who meets both the clinical and epidemiological criteria, while a confirmed case is any individual who meets both the clinical and laboratory criteria.

### 2.3. Serologic Testing

Serum samples were tested for specific anti-CCHFV IgM and IgG antibodies using commercial enzyme-linked immunosorbent assay (ELISA) kits. Between 2015 and 2022, VectoCrimean-CHF-IgG and VectoCrimean-CHF-IgM kits (Vector-Best, Koltsovo, Russia) were used. From 2023 onwards, Anti-Crimean–Congo fever virus (CCHFV) ELISA (IgG) and Anti-Crimean–Congo fever virus (CCHFV) ELISA (IgM) kits (Euroimmun, Lübeck, Germany) were adopted. Each sample was tested using a single kit, based on availability at the time. All procedures were performed according to the respective manufacturers’ instructions.

### 2.4. RNA Extraction

Viral RNA extraction was performed using either the QIAamp^®^ Viral RNA Mini Kit (QIAGEN, Hilden, Germany) or PureLink^®^ Viral RNA/DNA Mini Kit (Invitrogen, Carlsbad, CA, USA) commercial kits, depending on availability. RNA was extracted using each kit according to the respective manufacturers’ instructions and stored at −80 °C before testing.

### 2.5. Real-Time Reverse Transcriptase Polymerase Chain Reaction (RT-qPCR)

For the detection of CCHFV, several TaqMan RT-qPCR protocols were employed: the assay developed by Garrison et al. [23]; the assay by Atkinson et al. [24]; and a commercial kit (Crimean–Congo Hemorrhagic Fever Virus Real-Time PCR Detection Kit, Viasure, Zaragoza, Spain). The QuantiTect^®^ Probe RT-PCR Kit (QIAGEN, Hilden, Germany) and SuperScript™ III Platinum^®^ One-Step qRT-PCR Kit (Invitrogen, Carlsbad, CA, USA) master mixes were used. The selection of reagents was guided by availability and certain experimental considerations. For example, certain one-step qRT-PCR master mixes were more compatible or validated with particular primer sets (e.g., those described by Garrison et al. [23] or Atkinson et al. [24]) and some reagents performed more reliably on specific instruments (ABI 7300 and Gentier96E real-time PCR systems). All reactions were carried out according to the respective manufacturers’ protocols.

### 2.6. Whole-Genome Sequencing

Next-generation sequencing was carried out using the Viral Surveillance Panel v2 on the Illumina MiSeq platform with the MiSeq V3 reagent kit (2 × 300 bp) (Illumina, San Diego, CA, USA). Only RT-qPCR–positive samples with a cycle threshold (Ct) value below 30 were selected for sequencing.

### 2.7. Bioinformatic Analysis

The quality of raw sequencing data was assessed using FastQC v0.12.1 (Babraham Institute, Cambridge, UK; https://www.bioinformatics.babraham.ac.uk/projects/fastqc/, accessed on 19 June 2025). The trimming and filtering of the raw reads were performed using fastp v0.23.4 (Shifu Chen, Shenzhen, China) [25]. Processed reads were mapped against the reference genome sequences for each segment (GenBank accession numbers: NC_005301.3 for L, NC_005300.2 for M, and NC_005302.1 for S), and consensus sequences were generated using Geneious Prime^®^ 2025.1.2 (Biomatters Ltd., Auckland, New Zealand; https://www.geneious.com/, accessed on 19 June 2025) with default parameters. The resulting segments were annotated with Vigor4 (JCVI, Rockville, MD, USA) [26].

Phylogenetic trees for each segment (L, M, and S) were constructed using the “non-linking” CCHFV Nextstrain build [27] available from the GitHub repository at https://github.com/neherlab/CCHFV, accessed on 19 June 2025. The pipeline downloads all sequences with available metadata that meet the minimum length criteria (S segment—1000 bp, M segment—4000 bp, and L segment—10,000 bp). For strain BG111-2024, which had an incomplete L segment, a gapped sequence was generated with Ns inserted at unresolved positions (~1.6% of the sequence). Reference sequences used for rooting were NC_005301.3 for segment L, NC_005300.2 for segment M, and NC_005302.1 for segment S. The resulting phylogenies were visualized using Auspice v2.62.0 (Nextstrain project, Seattle, WA, USA) [28].

### 2.8. Data Availability

The sequences of the two CCHFV strains analyzed in this study have been deposited in GenBank. Each strain includes three genomic segments (L, M, and S). Complete sequences for all three segments (L, M, and S) have been submitted for strain BG121-2016. For strain BG111-2024, the S and M segments were complete, while the L segment was uploaded as partial. The GenBank accession numbers are as follows:

BG121-2016: L segment-PV696552; M segment-PV696550; S segment-PV696548.

BG111-2024: L segment-PV696553; M segment-PV696551; S segment-PV696549.

## 3. Results

### 3.1. Epidemiological Characteristics

**Figure 1:**
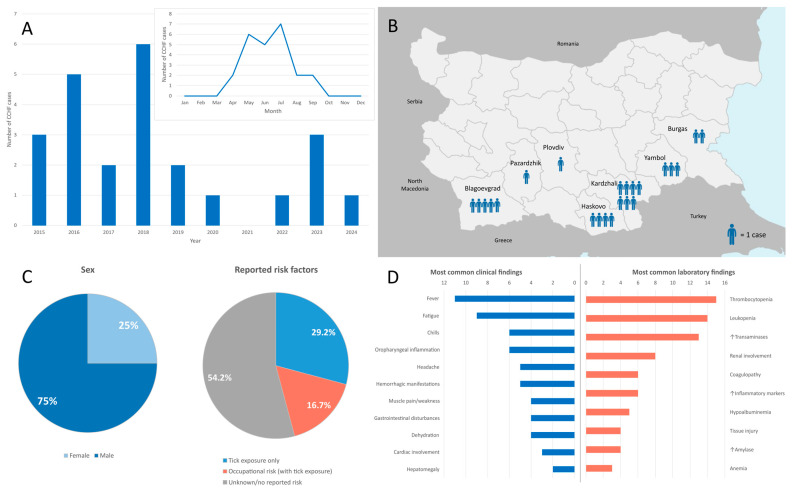
Summary of epidemiological, clinical and laboratory characteristics of CCHF cases in Bulgaria, 2015–2024. (**A**) Distribution of cases by year and by month. (**B**) Geographic distribution of cases by province. (**C**) Reported exposures among patients with available epidemiological data. (**D**) Frequency of clinical and laboratory findings among patients with available clinical and paraclinical data.

Between 2015 and 2024, a total of 24 human cases with laboratory-confirmed Crimean–Congo hemorrhagic fever (CCHF) were identified. The distribution of cases by year was as follows: 2015 (*n* = 3, 12.5%), 2016 (*n* = 5, 20.8%), 2017 (*n* = 2, 8.3%), 2018 (*n* = 6, 25%), 2019 (*n* = 2, 8.3%), 2020 (*n* = 1, 4.2%), 2021 (*n* = 0, 0%), 2022 (*n* = 1, 4.2%), 2023 (*n* = 3, 12.5%) and 2024 (*n* = 1, 4.2%). All cases occurred during the warmer months: April (*n* = 2, 8.3%), May (*n* = 6, 25%), June (*n* = 5, 20.8%), July (*n* = 7, 29.2%), August (*n* = 2, 8.3%) and September (*n* = 2, 8.3%) (Figure 1A, Supplementary Table S1).

The highest number of cases occurred in the province of Kardzhali (*n* = 7, 29.2%), followed by Blagoevgrad (*n* = 5, 20.8%), Haskovo (*n* = 4, 16.7%), Yambol (*n* = 3, 12.5%), Burgas (*n* = 2, 8.3%) and one case each from Pazardzhik and Plovdiv (4.2%) (Figure 1B). For one patient, the geographic location of exposure was not recorded.

The majority of patients were male (*n* = 18, 75%), and six were female (25%). The patients’ ages ranged from 14 to 78 years, with a median of 62.5 years and an interquartile range (IQR) of 23.5 years. Eleven individuals (45.8%) reported tick exposure, including four (16.7%) with known occupational risk factors (animal herders, a farmer, and a forest worker) (Figure 1C).

### 3.2. Clinical Findings

Among the 13 patients with available clinical data, initial symptoms commonly included fever (*n* = 8, 61.5%), fatigue (*n* = 7, 53.8%), chills (*n* = 5, 38.5%), headache (*n* = 4, 30.8%) and myalgia (*n* = 3, 23.1%). Gastrointestinal disturbances like nausea, vomiting, abdominal pain and diarrhea were also observed, as well as dizziness, arthralgia, and anorexia. One person had reported hematoma on their left arm, suggesting early coagulopathy.

On hospital admission, the majority of patients were in an impaired to moderately impaired general condition with varying degrees of dehydration. Hepatic involvement was clinically evident in two patients with palpable hepatomegaly, while the remainder had no hepatosplenomegaly on physical examination. Hemorrhagic manifestations were observed in five patients and included petechial lesions on the lower limbs, bleeding gums, and melena. Epistaxis, hematuria, enanthem of the oral mucosa, hematemesis and hematomas at venipuncture sites were also observed. Hemorrhagic symptoms were absent in eight patients. Other clinical signs included conjunctival injection, oropharyngeal inflammation and cardiac and pulmonary manifestations (Figure 1D, Table S1).

### 3.3. Fatal Cases

Of the 24 patients with confirmed CCHF, 2 fatalities (8.3%) were recorded. The first fatal case was a 76-year-old woman from Blagoevgrad who fell ill in July 2015. She tested positive for CCHF-specific IgM antibodies. The second case was a 68-year-old woman from Plovdiv who became infected in September 2018 and had a history of tick exposure. Diagnosis was confirmed by the detection of serum IgM antibodies. No additional data are available for these patients.

### 3.4. Laboratory Findings

The laboratory data related to routine clinical assessment were available for 16 patients. Leukopenia (*n* = 14, 87.5%) and thrombocytopenia (*n* = 15, 93.75%) were observed in almost all patients. For one of these sixteen patients, laboratory data on platelet count were missing. Coagulopathy was evident in six patients (37.5%), characterized by an elevated international normalized ratio (INR), prolonged prothrombin time (PT) and activated partial thromboplastin time (APTT), reduced PT activity, low fibrinogen levels and elevated D-dimer, indicating varying degrees of clotting dysfunction and fibrinolytic activity. Liver dysfunction was documented in the majority of patients (*n* = 13, 81.3%), mostly with elevated transaminases (alanine aminotransferase [ALT] and aspartate aminotransferase [AST]), as well as increased gamma-glutamyl transferase (GGT), alkaline phosphatase (ALP) and bilirubin in some patients. A few patients had hypoalbuminemia and low total protein, indicating hepatic synthetic impairment. Renal involvement was observed in several cases (*n* = 8, 50%), manifesting most often as proteinuria, hematuria and glucosuria. Hypokalemia, hypochloremia and hyperglycemia were also present in some cases. Markers of inflammation (the C-reactive protein [CRP], the erythrocyte sedimentation rate [ESR]) and tissue injury (lactate dehydrogenase [LDH], creatine kinase [CK], and CK-MB) were frequently elevated, suggesting widespread cellular damage and systemic inflammation. Pancreatic involvement, through elevated serum amylase, was also evident in several patients (Figure 1D, Table S2).

### 3.5. CCHF Laboratory Results

Of the 24 patients included in this study, 7 (29.2%) had positive RT-qPCR results with only two of the positive samples having Ct values below 30 (Supplementary Table S2). CCHFV-specific IgM antibodies were detected in 23 patients (95.8%). Six patients (25%) had both positive RT-qPCR and positive IgM results. CCHFV-specific IgG antibodies were found at initial testing in one patient (4.2%). In follow-up samples, IgG antibodies were found present in ten patients (41.2%).

### 3.6. Phylogenetic Analysis

Complete S, M, and L segment sequences were obtained for strain BG121-2016. For strain BG111-2024, the S and M segments were complete, while the L segment was partial, with approximately 1.6% ambiguous bases (Ns). The first strain (BG121-2016) was derived from a 62-year-old male patient in Kardzhali, who became ill in September 2016. The second strain (BG111-2024) was isolated from a 14-year-old male in Blagoevgrad, diagnosed in August 2024. 

**Figure 2:**
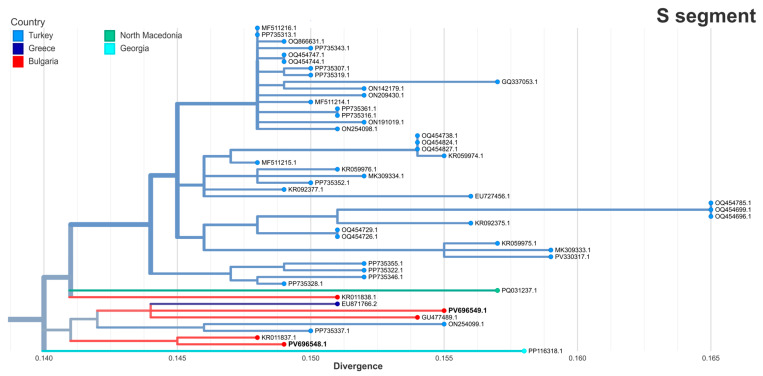
The maximum likelihood phylogeny of the S segment of Crimean–Congo hemorrhagic fever virus (CCHFV). A focused view of the Europe 1 lineage (clade V) containing the two Bulgarian strains from this study (in bold) is shown with branch tips colored by country of origin. The full clade is not displayed.

Phylogenetic analysis revealed that the S segments from both strains belonged to the Europe 1 genotype (clade V) (Figure 2). Both strains clustered within the Turkish branch together with previously reported Bulgarian isolates, including the Bulgarian vaccine strain GU477489.1, originally isolated from a patient in 1981 [29]. The S segment of strain BG121-2016 (PV696548.1) clustered most closely with a 2013 human strain from Haskovo, Bulgaria (KR011837.1, 99.27% identity). The S segment of strain BG111-2024 (PV696549.1) was most closely related to a strain from Greece (EU871766.2, 98.5% identity) obtained in 2008 from a patient living near the border with Bulgaria [30].

**Figure 3:**
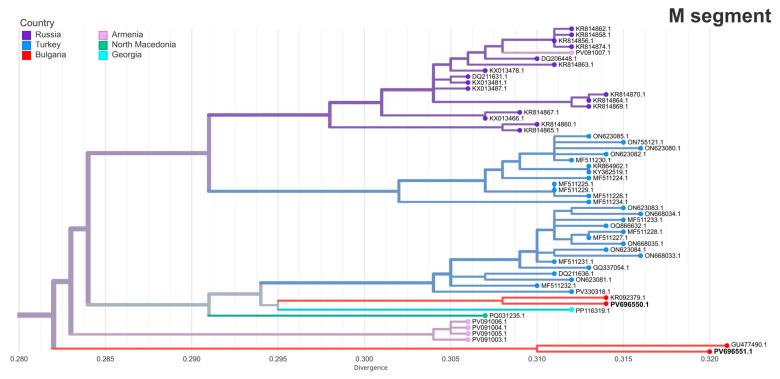
The maximum likelihood phylogeny of the M segment of Crimean–Congo hemorrhagic fever virus (CCHFV). A focused view of the clade containing the two Bulgarian strains from this study (in bold) is shown with branch tips colored by country of origin. The full clade is not displayed.

The M segments of the two strains in this study were located in a distinct cluster composed primarily of Turkish and Russian strains, as well as strains from Armenia, North Macedonia and Georgia (Figure 3). PV696550.1 (BG121-2016) shared its sub-branch with Turkish isolates and showed the highest similarity with another Haskovo strain from 2013 (KR092379.1, 98.91% identity). PV696551.1 (BG111-2024) was most closely related to the Bulgarian vaccine strain (GU477489.1, 98.32% identity).

**Figure 4:**
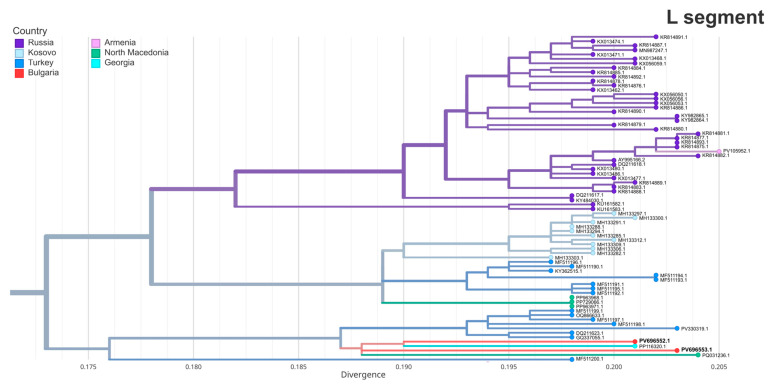
The maximum likelihood phylogeny of the L segment of Crimean–Congo hemorrhagic fever virus (CCHFV). A focused view of the clade containing the two Bulgarian strains from this study (in bold) is shown with branch tips colored by country of origin. The full clade is not displayed.

An analysis of the L segments of the two strains placed them in a distinct Eastern European/Balkan cluster (Turkish branch) together with strains from Turkey, North Macedonia and Georgia (Figure 4). PV696552.1 (BG121-2016) showed the highest similarity to a 2023 Georgian strain from a human spleen (PP116320.1, 97.85% identity). PV696553.1 (BG111-2024) was most closely related to a 2024 strain from North Macedonia (PQ031236.1, 97.5% identity, 96% query coverage), collected from a human case in the northeast of the country, near the Bulgarian border [31].

Amino acid sequence analysis showed that both strains carried lysine (K) at the GPC 517 position (corresponding to position 516 in the original study), which has been associated with enhanced fusion and infectivity in vitro [32]. The K517 variant was conserved across all strains in the same cluster.

## 4. Discussion

Bulgaria is one of the few European countries where human CCHF cases are reported regularly—cases have been registered nearly every year between 1990 and 2024 [18], with the annual number generally ranging from 1 to 22 (exceptions include a peak of 54 cases in 2002 and an absence of reported cases in 2021). Seroprevalence studies in both humans [33] and livestock [34,35] further support the ongoing circulation of CCHFV, particularly in the southern and southeastern regions of the country. Infected ticks—especially *Hyalomma marginatum*—have also been documented in these areas, with one study reporting an infection rate of 6.3% [17]. Overall, this suggests an ongoing, localized viral circulation in Bulgaria’s border regions with Turkey and Greece, which was also reflected in the case distribution observed in this study—the highest number of cases were reported from Kardzhali, Blagoevgrad, Haskovo and Yambol.

Several factors contribute to the concentration of CCHF cases in the southern and southeastern regions of Bulgaria. These regions have the highest concentrations of cattle and sheep farming in the country [36], which, together with the presence of wildlife such as hares and hedgehogs, provide ample hosts for tick populations. Climatic conditions in these areas, characterized by milder winters and warmer temperatures, favor the survival and reproduction of *Hyalomma* ticks, leading to higher tick densities and extended periods of activity [37]. Additionally, the presence of open grasslands and shrublands offers ideal habitats for these ticks [37]. 

The demographic profile of cases in this study is consistent with earlier studies with most patients being adult men, likely reflecting occupational exposure risks such as farming or animal handling [8,38,39]. Only one pediatric case was reported, which aligns with previous observations that CCHF in children may be milder and therefore less frequently diagnosed [40,41]. Clinical features were consistent with previous reports [40,42,43], with fever, fatigue, chills, headache, gastrointestinal symptoms, and hemorrhagic signs being most commonly reported. Similarly, the laboratory findings—thrombocytopenia, leukopenia, elevated transaminases, coagulopathy, increased LDH, and inflammatory markers—were similar to previous descriptions of CCHF cases [1,2,44]. The laboratory confirmation of CCHFV infection in all patients was based on detection of CCHFV RNA or anti-CCHFV IgM antibodies. A subset of patients was positive for both, likely reflecting a transitional phase of infection in which viremia is declining but still detectable (Ct values >30 in most cases), while the host immune response has already been initiated. Viremia generally starts declining several days after onset of illness [45,46,47] and the short window during which it can be detected highlights the importance of combining molecular and serological methods to improve diagnosis.

The phylogenetic analysis of the two sequenced strains in this study grouped them together with strains previously detected in Bulgaria and neighboring countries. Although the two strains were sampled eight years apart and from distinct geographic locations, they both clustered within the Turkish branch of the Europe 1 genotype (clade V). While minor differences were observed, the sequences were consistently clustered within the same phylogenetic group across all three segments, making reassortment events unlikely. The high genetic similarity of the strains in this study with past Bulgarian strains, including the historical Bulgarian vaccine strain from 1981, suggest that the same lineage has likely remained endemic in Bulgaria for decades. The persistent circulation of a relatively stable CCHFV lineage in the Balkan peninsula may be a reflection of the enzootic nature of CCHFV in the region, where viral transmission is sustained with minimal external introduction. Both strains carried the conserved K517 variant in the glycoprotein precursor, a substitution associated with enhanced viral fusion and infectivity in vitro [32], which may contribute to the evolutionary stability of this lineage. Cross-border genetic similarities, particularly between the 2024 strain and isolates from Greece [30] and North Macedonia [31], might indicate shared ecological dynamics and the unrestricted movement of infected ticks and animals. The 2024 strain originated from Blagoevgrad, a region situated near the borders with both Greece and North Macedonia, which further supports the possibility of viral dissemination between neighboring countries. The 2016 strain was derived from a patient in Kardzhali, a province in close proximity to both Turkey and Haskovo, which may explain its high similarity to previously reported strains from those regions.

Limitations of this study include the relatively small number of cases with complete clinical, laboratory and epidemiological data, the retrospective nature of data collection and the absence of information on patient treatment. Virus isolation in cell culture was not performed due to limited laboratory capacity. Sequencing was available only for two cases and the full genetic diversity of CCHFV cannot be captured. Continued sequencing is essential to detect potential shifts in viral genetic makeup and identify emerging variants.

Despite these limitations, this study provides information about the clinical spectrum and epidemiology of CCHFV in Bulgaria. The detection of genetically similar, regionally clustered strains suggests a well-established local viral transmission within Bulgaria and the Balkan region. Combining molecular surveillance with clinical and epidemiological data remains essential for improving public health preparedness and for deeper understanding of CCHFV evolution and persistence in endemic regions.

## Data Availability

The full-length gapped L segment sequence of strain BG111-2024 is available from the authors upon request.

## Supplementary Materials

The following supporting information can be downloaded at https://www.mdpi.com/article/10.3390/pathogens14080785/s1, Table S1. Epidemiological and clinical characteristics of laboratory-confirmed Crimean–Congo hemorrhagic fever (CCHF) cases in Bulgaria, 2015–2024. Table S2. Laboratory findings in confirmed Crimean–Congo hemorrhagic fever in Bulgaria, 2015–2024.

## Abbreviations

The following abbreviations are used in this manuscript:

CCHF	Crimean–Congo hemorrhagic fever
CCHFV	Crimean–Congo hemorrhagic fever virus
ELISA	Enzyme-linked immunosorbent assay
RT-qPCR	Real-time reverse transcriptase polymerase chain reaction
GPC	Glycoprotein precursor
